# Words hurt: common and distinct neural substrates underlying nociceptive and semantic pain

**DOI:** 10.3389/fnins.2023.1234286

**Published:** 2023-09-27

**Authors:** Eleonora Borelli, Francesca Benuzzi, Daniela Ballotta, Elena Bandieri, Mario Luppi, Cristina Cacciari, Carlo Adolfo Porro, Fausta Lui

**Affiliations:** ^1^Department of Medical and Surgical Sciences, University of Modena and Reggio Emilia, Modena, Italy; ^2^Department of Biomedical, Metabolic and Neural Sciences, University of Modena and Reggio Emilia, Modena, Italy; ^3^Oncology and Palliative Care Units, Civil Hospital Carpi, USL, Carpi, Italy; ^4^Hematology Unit and Chair, Azienda Ospedaliera Universitaria di Modena, Modena, Italy

**Keywords:** pain, semantics, language, words, social pain-related words, physical pain-related words, nociception, fMRI

## Abstract

**Introduction:**

Recent studies have shown that processing semantic pain, such as words associated with physical pain, modulates pain perception and enhances activity in regions of the pain matrix. A direct comparison between activations due to noxious stimulation and processing of words conveying physical pain may clarify whether and to what extent the neural substrates of nociceptive pain are shared by semantic pain. Pain is triggered also by experiences of social exclusion, rejection or loss of significant others (the so-called social pain), therefore words expressing social pain may modulate pain perception similarly to what happens with words associated with physical pain. This event-related fMRI study aims to compare the brain activity related to perceiving nociceptive pain and that emerging from processing semantic pain, i.e., words related to either physical or social pain, in order to identify common and distinct neural substrates.

**Methods:**

Thirty-four healthy women underwent two fMRI sessions each. In the Semantic session, participants were presented with positive words, negative pain-unrelated words, physical pain-related words, and social pain-related words. In the Nociceptive session, participants received cutaneous mechanical stimulations that could be either painful or not. During both sessions, participants were asked to rate the unpleasantness of each stimulus. Linguistic stimuli were also rated in terms of valence, arousal, pain relatedness, and pain intensity, immediately after the Semantic session.

**Results:**

In the Nociceptive session, the ‘nociceptive stimuli’ vs. ‘non-nociceptive stimuli’ contrast revealed extensive activations in SI, SII, insula, cingulate cortex, thalamus, and dorsolateral prefrontal cortex. In the Semantic session, words associated with social pain, compared to negative pain-unrelated words, showed increased activity in most of the same areas, whereas words associated with physical pain, compared to negative pain-unrelated words, only activated the left supramarginal gyrus and partly the postcentral gyrus.

**Discussion:**

Our results confirm that semantic pain partly shares the neural substrates of nociceptive pain. Specifically, social pain-related words activate a wide network of regions, mostly overlapping with those pertaining to the affective-motivational aspects of nociception, whereas physical pain-related words overlap with a small cluster including regions related to the sensory-discriminative aspects of nociception. However, most regions of overlap are differentially activated in different conditions.

## 1. Introduction

Translating the experience of pain into words is a challenge, as attested by scientific evidence, literary sources, and personal experience. Nonetheless, language remains the main medium for conveying our own experience of pain to others, including health professionals (Galli et al., 2019). The International Association for the Study of Pain (IASP) recently revised the definition of pain to “an unpleasant sensory and emotional experience associated with, or resembling that associated with, actual or potential tissue damage.” In an accompanying note, it is mentioned that “a person’s report of an experience as pain should be respected,” referring to the fact that the subjective nature of pain should not be interpreted as less valid or reliable (Raja et al., 2020). Pain is defined and ultimately evaluated by subjective reports: as Gracely (2016) put it, “Much can be inferred from objective measures of anatomy, physiology, and behavior, but verbal report remains the standard by which all other measures are compared.” This led to the use, in medical research, of questionnaires that should capture different aspects of the pain experience by asking patients to translate their pain into standardized pain descriptors (e.g., McGill Pain Questionnaire—MPQ; Melzack, 1975; Main, 2016).

Since pain communication significantly relies on language, it is important to establish how the mind and the brain treat the complex relationships between words and pain. Accumulating evidence suggests that actual physical pain (nociceptive pain) and the pain conveyed by words (semantic pain) influence each other at behavioral and neural levels (e.g., Knost et al., 1997; Dillmann et al., 2000; de Wied and Verbaten, 2001; Pincus and Morley, 2001; Weiss et al., 2003; Sitges et al., 2007; Wang et al., 2008; Chooi et al., 2011; Meng et al., 2012; Ott et al., 2012; Schoth et al., 2012; Crombez et al., 2013; Ritter et al., 2016; Schoth and Liossi, 2016; Swannell et al., 2016; Reuter et al., 2017; Brodhun et al., 2021; Borelli et al., 2021b). This evidence clearly shows that the experience of physical pain affects the way in which we process pain-related words, and that the presentation of pain-related words impacts on the experience of physical pain; therefore, we can consider language as part of the broad set of endogenous modulators (Koban et al., 2017; Seymour, 2019) which ultimately modulate the processing and perception of pain. However, despite an increasing number of studies, the neural architecture underlying the bi-directional relationships between language and pain is not yet fully understood.

The brain response to a nociceptive stimulus consists in the activation of a complex network of cortical and subcortical structures (Fauchon et al., 2020; Xu et al., 2020), commonly referred to as “pain matrix” (Ingvar, 1999; Singer et al., 2004; Tracey, 2005; Jääskeläinen and Kosonogov, 2023; Kumari et al., 2023). The pain matrix is thought to play a key role in elaborating two important aspects of the nociceptive experience: the sensory-discriminative aspect and the affective-motivational aspect (Melzack and Casey, 1968; for overviews, see Price, 2000; Auvray et al., 2010). The sensory-discriminative aspect is processed by the primary and secondary somatosensory cortices (SI and SII, respectively), and posterior insula, which are sometimes referred to as the “lateral component” of the pain matrix (because it projects through specific lateral thalamic nuclei; Treede et al., 1999); the affective-motivational aspect is processed by the anterior insula (AI) and the anterior mid-cingulate cortex (aMCC), in turn, sometimes referred to as the “medial component” of the pain matrix (because it projects through specific medial thalamic nuclei; Melzack and Casey, 1968; Treede et al., 1999; Kulkarni et al., 2005; Vogt, 2016). The thalamus is therefore involved in both the sensory-discriminative and the affective-motivational components, with prominent functions played by different nuclei in one or the other (Ab Aziz and Ahmad, 2006).

A handful of neuroimaging studies on healthy participants has shown that, in the absence of any noxious stimuli, the brain areas engaged in processing pain-related words partly overlap with those thought to be involved in experiencing physical pain, both the affective-motivational component of the pain matrix (Osaka et al., 2004; Kelly et al., 2007; Ritter et al., 2016), and also the sensory-discriminative one (Gu and Han, 2007; Richter et al., 2010).

Across many different languages, the words that describe physical pain are often used also to convey the so-called social pain, namely, the painful feelings associated with actual or potential social rejection, exclusion, or loss (e.g., betrayal can be described as a stab, a divorce as a scar, a defeat as being painful; Eisenberger and Lieberman, 2005; MacDonald and Leary, 2005).

These ways of referring to social pain are not simple metaphorical extensions borrowed from otherwise unrelated experiences of physical pain: according to the literature, physical and social pain are more neurally intertwined than it was initially thought (for an overview, see Eisenberger, 2015). This is not surprising, since social bonds are fundamental for survival in mammalians, and their interruptions represent a threat potentially as relevant as a noxious stimulus (MacDonald and Leary, 2005; Eisenberger, 2012). Lesion and neuroimaging studies have shown that physical and social pain partly share the same neural substrates, predominantly in the affective-motivational part of the pain matrix (e.g., Eisenberger et al., 2003, 2007; Cacioppo et al., 2013; Cristofori et al., 2013). In these studies, social pain was predominantly elicited through the participant’s exclusion in a virtual-ball game, the Cyberball game (Williams et al., 2000). In the Cyberball game, participants are led to believe that they are playing online with other real people, whereas they are actually playing against the computer. The game consists of throwing the ball at each other. The computer is programmed to initially include the participant in the game and then increase the ball exchanges between the other simulated players to exclude the participant. Exclusion in the Cyberball game is considered a form of ostracism, involving being ignored or excluded by others. It is considered a reliable paradigm to induce negative feelings of distress, decreased satisfaction of the need to belong, and other psychological responses associated with social exclusion. Since the affective-motivational pain component is crucial for signaling an aversive state and for motivating behaviors aimed to reduce or escape pain, the activation of this component was interpreted as a hallmark of the neural overlap of physical and social pain. Some studies on social pain also reported activation of sensory-related brain regions, especially when the neural underpinnings of physical and social pain were tested within the same individuals (Novembre et al., 2015) and/or with tasks and stimuli eliciting social pain more powerfully than with the standard version of the Cyberball game (e.g., by having participants, who recently experienced an unwanted break-up, viewing a photo of the ex-partner; Kross et al., 2011).

However, whether, and the extent to which, social pain operates on the same neural pain matrix as nociceptive inputs is still a matter of discussion (Somerville et al., 2006; Cacioppo et al., 2013; Perini et al., 2018; Mwilambwe-Tshilobo and Spreng, 2021; for an overview, see Rotge et al., 2015; but see also Eisenberger, 2015).

Notwithstanding the fact that social pain may also be conveyed by words (Zhang et al., 2019), it has predominantly been studied using either the Cyberball game or non-verbal stimuli reminiscent of socially painful experiences (Kross et al., 2007, 2011; Takahashi et al., 2009; Wager et al., 2009; Fisher et al., 2010; Eisenberger, 2012). Therefore, it is still an open question whether social pain-related words indeed are as powerful as visual images or virtual games in eliciting brain responses in the pain matrix.

The aim of the present study is threefold: (i) to compare the brain areas involved in experiencing nociceptive pain and in processing semantic pain conveyed by physical and social pain-related words in the same individuals; (ii) to clarify whether the processing of semantic pain as conveyed by either physical pain-related words or social pain-related words recruits common or different brain regions; and (iii) to define whether semantic pain activations only concern the affective-motivational dimension of pain or also the sensory-discriminative dimension. Finding involvement also of the sensory-discriminative dimension of pain would support the view that pain-related words resonate with past pain experiences, reactivating their memory, be they associated to physical or social events.

## 2. Materials and methods

### 2.1. Participants

Because of the well-documented gender differences on pain perception (Dai et al., 2018) and social exclusion perception (Benenson et al., 2013; Tomova et al., 2014; Morese et al., 2019), an all-female sample was preferred over a gender-mixed sample (Novembre et al., 2015; Benuzzi et al., 2018). Thirty-seven right-handed healthy females participated in the fMRI experiment after informed consent. Two were excluded because they did not accept to undergo the second fMRI session, and one was excluded because of a minor abnormal finding that emerged during the first structural scan. Therefore, the final sample was composed of 34 female participants (age range: 18–34 years, mean age: 22.6 years, SD = 3), which is considered an adequate sample size for fMRI analysis according to Friston (2012). Handedness was assessed by means of the Edinburgh Inventory (Oldfield, 1971). Inclusion criteria were to be Italian native speakers, with no history of psychiatric or neurological illness, no current or past condition of chronic pain, and no current use of any psychoactive medications. Participants were rewarded for their participation. The study was conducted according to the 2013 version of the Declaration of Helsinki and was approved by the Ethics Committee of Modena.

### 2.2. Personality assessment

In order to correlate the functional MR results with individual personality characteristics, participants were also presented with the Behavioral Inhibition and Behavioral Activation Scales (BIS/BAS; Carver and White, 1994; Italian version: Leone et al., 2002) and the Interpersonal Reactivity Index (IRI, formed by four subscales: Empathic Concern, Perspective Taking, Fantasy, Personal Distress; Davis, 1980; Italian version: Albiero et al., 2006). The activation and inhibition systems measured by the BIS/BAS are thought to play an important role in pain processing in both healthy individuals and chronic pain patients (Jensen et al., 2015; Serrano-Ibáñez et al., 2018). IRI was administered because of the several studies attesting the role of empathy in elaborating pain-related information (for an overview, see Xiang et al., 2018).

### 2.3. Stimuli

#### 2.3.1. Linguistic stimuli

Participants were visually presented with 102 Italian singular nouns belonging to the following categories: 51 positively-valenced nouns (henceforth, PosW; e.g., dono, present) and 51 negatively-valenced nouns, of which 17 not related to pain (henceforth, NegNoPW; e.g., immondizia, rubbish), 17 related to physical pain (henceforth, PhysPW; e.g., cefalea, cephalalgy), and 17 related to social pain (henceforth, SocPW; e.g., abbandono, abandonment). PhysPW and SocPW were selected from the Words Of Pain database, a normed collection of Italian pain words (WOP; Borelli et al., 2018), which also reports quantitative data on how much each word refers to a type of pain rather than the other, allowing us to avoid ambiguity in the selection process; PosW and NegNoPW were selected from the Italian version of the Affective Norms for English Words database (Italian ANEW; Montefinese et al., 2014). PosW were used as fillers to avoid a negativity bias potentially induced by an all-negative word experiment and were not discussed, while NegNoPW were used as a control condition (Richter et al., 2010), being pain defined as an unpleasant experience, i.e., associated to negative affect (Raja et al., 2020).

We chose the words in the different conditions so that they were balanced for the main psycholinguistic, distributional, affective, and pain-related variables that are known to influence comprehension processes, based on WOP and Italian ANEW scores. Specifically, PosW and the negatively-valenced words (i.e., NegNoPW, PhysPW, and SocPW all together) had, as expected, a significantly different valence (Mann–Whitney test; U = 0, *p* < 0.001) but were balanced for frequency (Mann–Whitney test; U = 1363.5, *p* = 0.68), length in letters (Mann–Whitney test; U = 1319.5, *p* = 0.9), familiarity (Student *t*-test; t = −0.220, *p* = 0.83), age of acquisition (Student *t*-test; t = 1.389; *p* = 0.17), imageability (Student *t*-test; t = 0.243; *p* = 0.81), concreteness (Mann–Whitney test; U = 1,461; *p* = 0.28), context availability (Student *t*-test; t = 1.793; *p* = 0.08), and arousal (Student *t*-test; t = 1.425; *p* = 0.16). NegNoPW, PhysPW, and SocPW were balanced for frequency (*F* = 0.588, *p* = 0.56), length in letters (*F* = 0.254, *p* = 0.78), familiarity (*F* = 1.399, *p* = 0.26), age of acquisition (*F* = 2.316, *p* = 0.11), imageability (*F* = 3.043, *p* = 0.06), context availability (*F* = 0.573, *p* = 0.57), valence (*F* = 0.566, *p* = 0.57), and arousal (*F* = 0.483, *p* = 0.62), but not for concreteness (*F* = 12.325, *p* < 0.001), with PhysPW significantly more concrete than both NegNoPW and SocPW (*p* = 0.007 and *p* < 0.001, respectively). PhysPW and SocPW were also balanced for pain intensity (Student *t*-test; t = 0.439, *p* = 0.66) and pain unpleasantness (Mann-Whitnet test; U = 123, *p* = 0.47), but SocPW were more pain-related (Student *t*-test; t = 2.597, *p* = 0.014). For the variables that are more strictly relevant for our research, i.e., valence, arousal, pain-relatedness, intensity, and unpleasantness, the words were balanced considering only the databases’ ratings obtained by females. Considering the inherent subjectivity in ratings, we also asked participants to rate each word for variables most relevant for the study (see section 2.4). The list of words is available as Supplementary Table 1.

#### 2.3.2. Mechanical stimuli

As in a prior study (Benuzzi et al., 2018), participants were administered cutaneous mechanical stimuli consisting of touching the skin with a sharp end (21 nociceptive stimulations, NocS) or with a rubber end (21 non-nociceptive stimulations, NonNocS; control condition) by means of a mechanical stimulator. The mechanical stimulator was custom-built in our laboratory and included four aluminum hollow cylinders, each one containing a sliding brass weight of 12, 25, 51, and 75 g, respectively. Each sliding brass weight ended with a plastic tip, on which a disposable stainless-steel wire (0.2 mm section) was mounted for nociceptive stimulation. A fifth aluminum hollow cylinder containing a sliding brass weight of less than 5 g ending with a foam-rubber tip (approximate diameter 2 mm) was mounted for tactile, non-nociceptive stimulation. The hollow cylinders had a lower-end opening that allowed the plastic tip and stainless-steel wire to protrude from the cylinder itself when it was maintained in a vertical orientation. The experimenter held the hollow cylinder vertically and perpendicular to the participant’s hand, with the stainless-steel wire positioned approximately 1 cm away from the skin. During each stimulation, triggered at pre-defined moments signaled by an LED, the experimenter gently lowered the hollow cylinder onto the hand. This action caused the tip to make contact with the skin and led the brass weight to slide up inside the hollow cylinder, transferring its weight onto the stainless-steel wire (see Supplementary Figure 1).

### 2.4. Procedures

**Figure 1:**
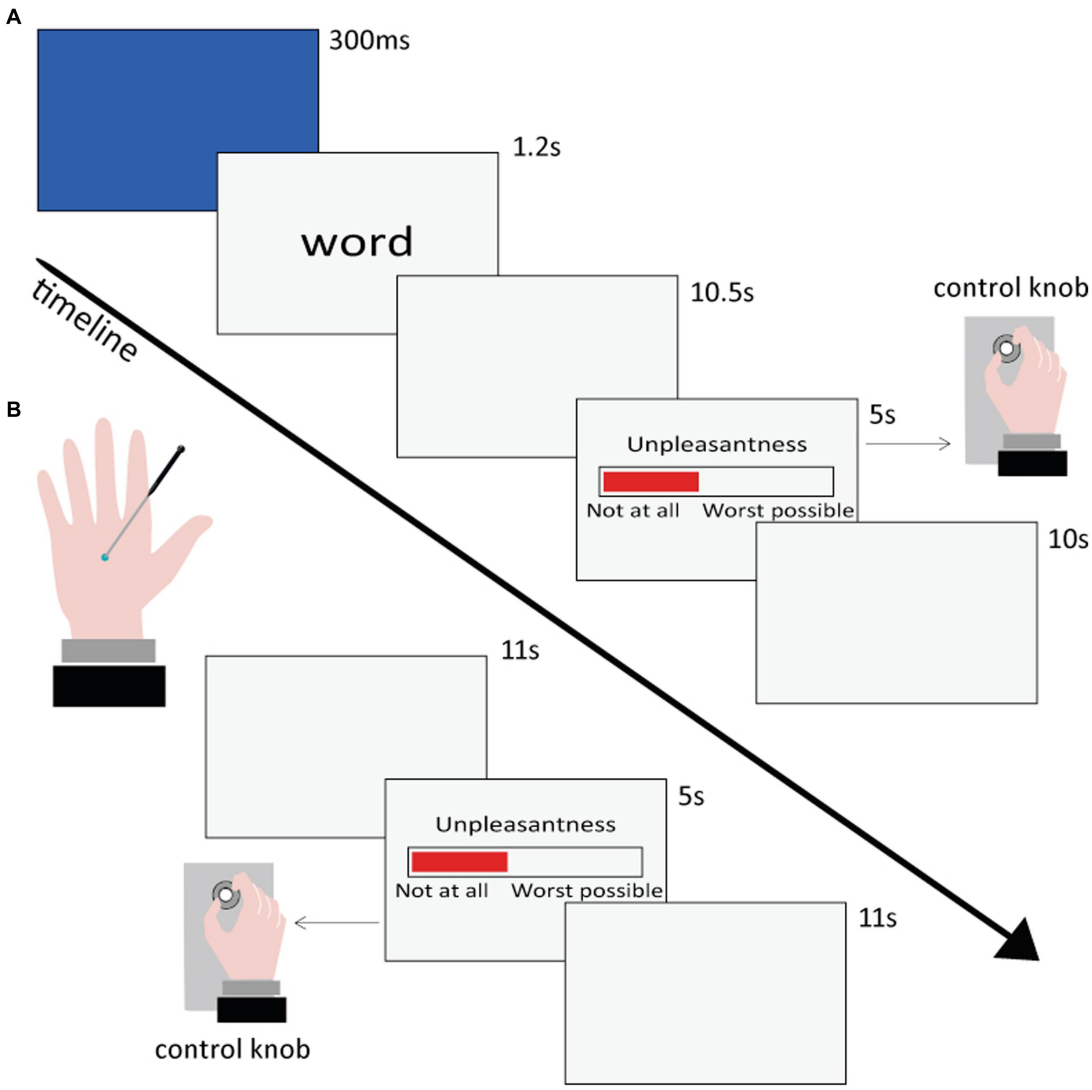
Time sequence of the presentation and rating of the stimuli in the Semantic **(A)** and Nociceptive **(B)** sessions.

All participants underwent two fMRI sessions (see Figure 1). In one session, they were visually presented with the linguistic stimuli (henceforth, Semantic session) and in the other session they received cutaneous mechanical stimulations (henceforth, Nociceptive session). The order of the Semantic and Nociceptive sessions was pseudo-randomized across participants. The interval between the two sessions ranged from a minimum of 2 days to a maximum of 2 weeks for each participant.

The Semantic session comprised four runs, each one lasting approximately 11 min. Within each run, 25 or 26 words were visually presented each in a separate trial in pseudo-random order (no more than three consecutive words belonging to the same category), so that all 102 words (see section 2.3.1) were presented once to each participant. Each trial began with a blue flash (300 ms) on the screen to capture the participant’s attention, followed by a lowercase word remaining at the center of the screen for 1.2 s. Participants were instructed to read the stimulus and wait for a Visual Analog Scale (VAS), which appeared on the screen after 10.5 s, and then rate the unpleasantness of the pain conveyed by each word by rotating a control knob previously fixed under their right hand. The extremes of the VAS scale were labeled as “Not unpleasant at all” and “Extremely unpleasant.” The VAS scale remained on the screen for 5 s. Once it disappeared, participants returned the knob to the initial position. In case of a rating equal to zero, participants were told to rapidly rotate the knob back and forth, in order to register a motor response also for words rated as not unpleasant at all. Finally, a gray screen followed for 10 s, and then the next trial started. The overall duration of each trial was 27 s.

Before the experiment started, the participants performed a few practice trials inside the scanner, which lasted approximately 2 min in total. Thus, the Semantic session including the practice trials lasted about 46 min. Stimulus presentation and response collection were carried out using an in-house built software developed in Visual Basic 6.^1^ The VAS ratings were converted into a 0–100 scale.

Once the Semantic session was concluded, participants were asked to rate, for each word: valence (on a Likert scale ranging from −3 to +3), arousal (on a Likert scale ranging from 1 to 7), pain-relatedness (on a Likert scale ranging from 1 to 7), and pain intensity (on a VAS scale ranging from “Not at all intense” to “The maximum imaginable intensity,” then converted into a 0–100 scale). These ratings were used to classify the words for the subsequent analysis and to carry out parametric analyses with functional data (see paragraph 2.6.2 fMRI data analyses).

The Nociceptive session comprised three runs, each lasting approximately 8 min. Within each run, 7 tactile stimuli and 7 painful stimuli were administered in a pseudo-random order (no more than three consecutive stimulations belonging to the same category). Tactile and painful stimuli were delivered to the dorsum of the left hand by means of a mechanical stimulator (see section 2.3.2). After each stimulation, participants were instructed to rate tactile and painful stimuli unpleasantness through the same procedure already described for the Semantic session. The overall duration of each trial was 27 s.

Before the experiment started, each participant’s pain threshold was set by means of a few tests, which lasted approximately 10 min in total. Thus, the Nociceptive session including the pain threshold measurement lasted about 34 min. As a result, brass weights were individually selected for each volunteer, so that the tip would induce a medium pain sensation (value of ~30–40 on the VAS scale) for painful stimuli and a pure tactile sensation (value of 0 on the VAS scale) for tactile stimuli. VAS ratings collection and transformation into 0–100 values were performed using the same in-house built software and procedure used for the Semantic session.

The in-house built software enabled experimenters to monitor both the precise stimulus timing and participants’ responses in both sessions in real-time from the control room; this ensured that there were no signs of fatigue, e.g., missing responses or decrease in accuracy.

### 2.5. MRI data acquisition and pre-processing

Functional MRI data were acquired by means of a Philips Achieva MRI system at 3 T and a BOLD (Blood Oxygenation Level Dependent)-sensitive gradient-echo echo-planar sequence [repetition time (TR): 2,000 ms; echo time (TE): 30 ms; field of view: 240 mm; 80 × 80 matrix; 35 transverse slices, 3 mm each with a 1 mm gap]. Each subject underwent four runs (348 or 361 volumes each, depending if 25 or 26 stimuli were presented in the run) for the Semantic session, and three runs (218 volumes each) for the Nociceptive session. A high-resolution T1-weighted anatomical image was also acquired for each participant to allow anatomical localization and spatial standardization (TR: 9.9 ms; TE: 4.6 ms; 170 sagittal slices; voxel size: 1 mm × 1 mm × 1 mm).

Processing of the functional images and statistical analyses were performed using Matlab 8.1.0.604 (The MathWorks Inc., Natick, MA, United States) and SPM12 softwares (Wellcome Department of Imaging Neuroscience, London, United Kingdom, https://www.fil.ion.ucl.ac.uk/spm/). Functional volumes of each participant were corrected for slice-time acquisition differences, realigned to the first volume acquired, normalized to the MNI (Montreal Neurologic Institute) template implemented in SPM12, and smoothed with a 9 × 9 × 12 mm FWHM Gaussian kernel.

### 2.6. Statistical analysis

#### 2.6.1. Behavioral data analyses

Descriptive (mean, standard deviation) and inferential statistics (ANOVAs and post-hoc tests for significant interactions) were computed on valence, arousal, pain-relatedness, and intensity ratings given by participants after the Semantic session and on the unpleasantness ratings given by participants during the Semantic session, for each category of semantic stimuli (PosW, NegNoPW, PhysPW, SocPW).

Descriptive statistics (mean, standard deviation) were also performed on scores of the BIS/BAS and IRI scales.

#### 2.6.2. fMRI data analyses

Eleven out of the 136 overall runs (8.1%) from the Semantic sessions and 3 out of the 102 overall runs (2.9%) from the Nociceptive sessions were discarded because of excessive movements during scanning.

Two different analyses were performed for each subject, one for the Semantic session and one for the Nociceptive session. The conditions of the Semantic session (NegNoPW, PhysPW, SocPW as conditions of interest, plus the PosW) and the two conditions of the Nociceptive session (NocS, NonNocS) were modeled by convolving the respective stimulus timing vectors with the standard hemodynamic response function. Condition effects were estimated using a general linear model framework, and region-specific effects were investigated with linear contrasts comparing the three conditions of interest of the Semantic session and the two experimental conditions of the Nociceptive session. For each volunteer, stimuli in the Semantic session were classified according to the valence ratings that the subject provided during the post-scanning session, whereas stimuli in the Nociceptive session were classified according to the unpleasantness ratings provided during scanning. The words that each participant rated as zero in valence were included in a “Neutral words” category (on average 15 words per participant), the words that were missed or unknown to the participant were included in an “Other words” category (on average 2 words per participant); these two categories were included in the analysis matrix, but were not further considered. The classification of words based on participants’ ratings differed by one word on average compared to the classification based on the WOP and ANEW databases, therefore this procedure did not cause a significant unbalance between the stimuli categories. Group random-effects analyses were performed by entering the individual contrast images corresponding to the effects of interest into separate one-sample t-tests.

In order to verify whether the areas involved in experiencing nociceptive pain mediate also the comprehension of physical and social pain-related words, the functional results of the NocS > NonNocS contrast were used as “spatial localizer” for the results of the Semantic session, and both qualitative and quantitative analyses were performed. Qualitatively, the two fMRI sessions were compared overlaying the functional blobs of the NocS > NonNocS, PhysPW > NegNoPW, and SocPW > NegNoPW contrasts on the standard T1 weighted brain template implemented in SPM12. A quantitative analysis was conducted using the thresholded image of the NocS > NonNocS contrast, to mask inclusively the results of the PhysPW > NegNoPW, and SocPW > NegNoPW contrasts.

Moreover, our results were compared with a recent meta-analysis (Jensen et al., 2016). Specifically, we verified which coordinates associated with noxious stimulation in healthy subjects were encompassed within, or in the immediate vicinity of (i.e., no more than 10 mm from) one of the regions we identified by means of the masking procedures. These coordinates were used for an additional Regions of Interest (ROI) analysis. With this method, we identified 12 ROIs (xyz coordinates are expressed in MNI throughout the paper): the anterior (xyz = 2, 36, 18) and middle cingulate cortex (xyz = 4, 12, 38), the right and left AI (xyz = 40, 14, 2 and xyz = −38, 10, 4, respectively), the right and left thalamus (xyz = 14, −14, 6 and xyz = −12, −12, 4, respectively), right putamen (xyz = 20, 10, −4), left parahippocampal gyrus (xyz = −26, 2, −14), the right and left cerebellum (xyz = 28, −64, −30, xyz = −36, −58, −34 and xyz = −34, −70, −22), and the postcentral gyrus (xyz = −58, −22, 18). The latter only was found within the masking of the PhysPW > NegNoPW contrast. It is to be noted that for most of the ROIs we followed the nomenclature proposed by Jensen et al. (2016), however, one of the foci (xyz = 4, 12, 38) these authors called simply cingulate actually falls within the aMCC according to the topography suggested by Vogt, 2016 (aMCC; see Figure 1B in Vogt, 2016, for coordinates of anterior cingulate cortex (ACC)-aMCC borders); therefore, although Jensen et al., 2016 never mention aMCC, we named this focus accordingly. ROIs were built as 6-mm radius spheres by means of the MarsBar function of SPM12.^2^ The beta values were extracted from each ROI and for each contrast of interest (NocS > NonNocS, PhysPW > NegNoPW, SocPW > NegNoPW); 12 different one-way repeated-measure ANOVAs were run, one for each ROI.

Finally, regression analyses were performed on participants’ BIS/BAS and IRI scores, whereas participants’ ratings of valence, arousal, pain-relatedness, intensity, and unpleasantness were used for parametric analyses.

A family-wise error (FWE) correction or a double statistical threshold (single-voxel statistics and spatial extent) were used; the latter allows to achieve a combined experiment-wise (i.e., corrected for multiple comparisons) significance level of α < 0.05, using the 3dClustSim AFNI routine,^3^ with the “-acf” option.

## 3. Results

### 3.1. Behavioral results

**Figure 2:**
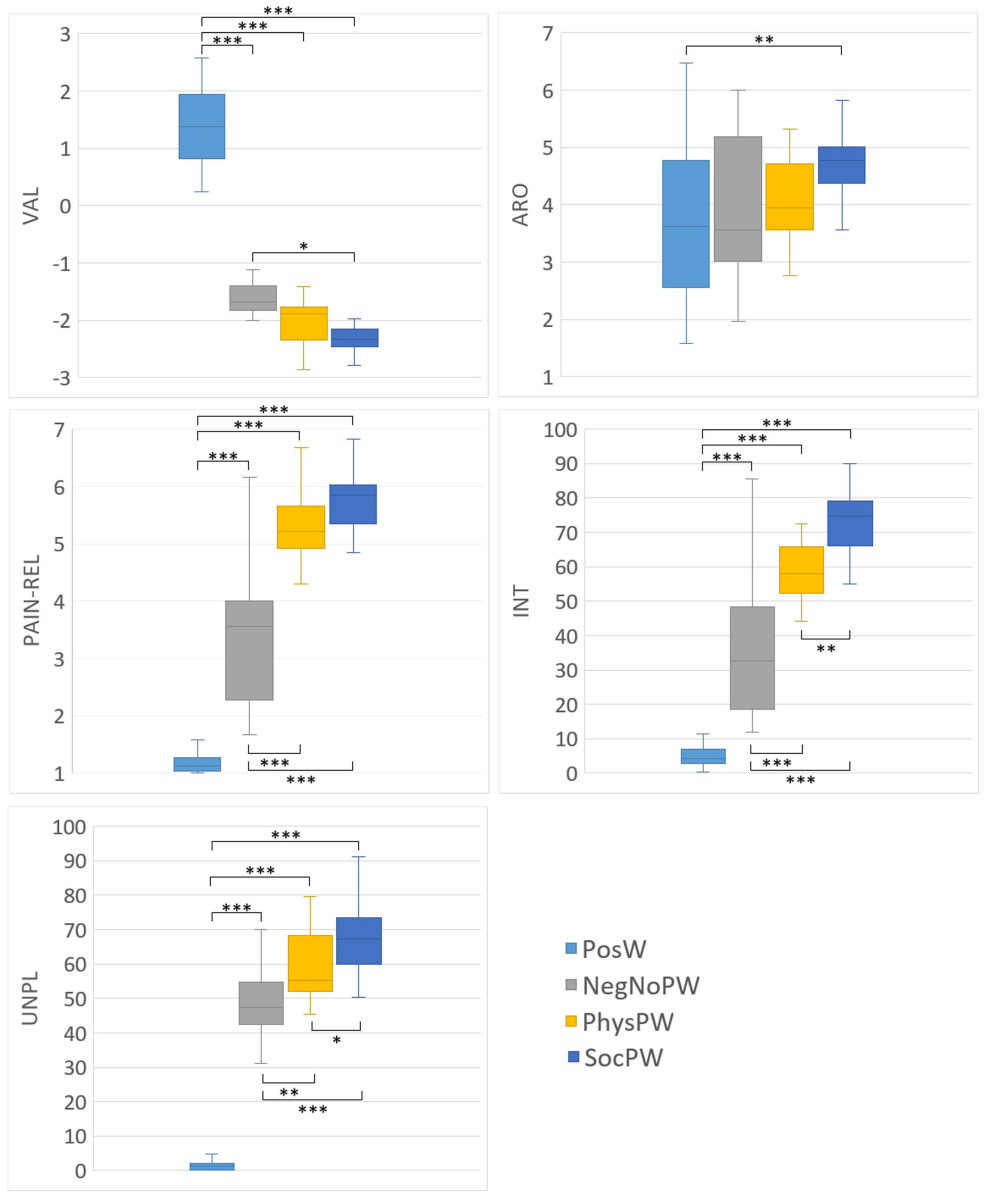
Box-and-whisker plots of the word ratings for valence, arousal, pain-relatedness, and intensity given by participants after the Semantic session, and of the word ratings for unpleasantness given by participants during the Semantic session, for each category of words (PosW, NegNoPW, PhysPW, and SocPW). Lower and upper lines represent 10th and 90th percentiles, respectively. Line inside the box represents the median. Lines connecting box-plots indicate significant differences with *p*-values of * < 0.05, ** < 0.01, and *** < 0.001. PosW, positive words; NegNoPainW, negative pain unrelated words; PhysPW, physical pain words; SocPW, social pain words; VAL, valence; ARO, arousal; PAIN-REL, pain relatedness; INT, intensity; UNPL, unpleasantness.

Descriptive statistics of the word ratings of valence, arousal, intensity, and pain-relatedness given by participants after the Semantic session, and of the word ratings of unpleasantness given by participants during the Semantic session, are reported in Supplementary Table 2 and plotted in Figure 2 for each category of words. Descriptive statistics of the BIS/BAS and IRI scores are reported in Supplementary Table 3.

As expected based on our experimental paradigm and stimulus selection, ANOVAs on participants’ word ratings of affective and pain-related variables revealed a significant difference between PosW, NegNoPW, PhysPW, and SocPW for valence (F_Brown-Forsythe_ = 467.648, *p* < 0.001), with PosW significantly more positive than NegNoPW, PhysPW, and SocPW (all *p* < 0.001); for pain relatedness (F_Brown-Forsythe_ = 199.353, *p* < 0.001), with PosW significantly less pain related than NegNoPW, PhysPW, and SocPW (all *p* < 0.001) and NegNoPW significantly less pain related than both PhysPW and SocPW (all *p* < 0.001); for intensity (F_Brown-Forsythe_ = 137.152, *p* < 0.001), with PosW conveying a significantly less intense pain than NegNoPW, PhysPW, and SocPW (all *p* < 0.001) and NegNoPW conveying a significantly less intense pain than PhysPW and SocPW (all *p* < 0.001); and for unpleasantness (F_Brown-Forsythe_ = 252.416, *p* < 0.001), with PosW conveying a significantly less unpleasant pain than NegNoPW, PhysPW, and SocPW (all *p* < 0.001) and NegNoPW conveying a significantly less unpleasant pain than PhysPW and SocPW (*p* = 0.003 and *p* < 0.001, respectively).

However, ANOVAs on participants’ word ratings of affective and pain related variables also revealed some unexpected differences. Specifically, they revealed a significant difference between PosW, NegNoPW, PhysPW, and SocPW for valence (F_Brown-Forsythe_ = 467.648, *p* < 0.001), with SocPW significantly more negative than NegNoPW (*p* = 0.011); for arousal (F_Brown-Forsythe_ = 5.084, *p* = 0.003), with SocPW significantly more arousing than PosW (*p* = 0.007); and for intensity (F_Brown-Forsythe_ = 137.152, *p* < 0.001) and unpleasantness (F_Brown-Forsythe_ = 252.416, *p* < 0.001), with SocPW conveying a significantly more intense and unpleasant pain than PhysPW (*p* = 0.006 and *p* = 0.048, respectively).

### 3.2. fMRI results

#### 3.2.1. Nociceptive session

**Figure 3:**
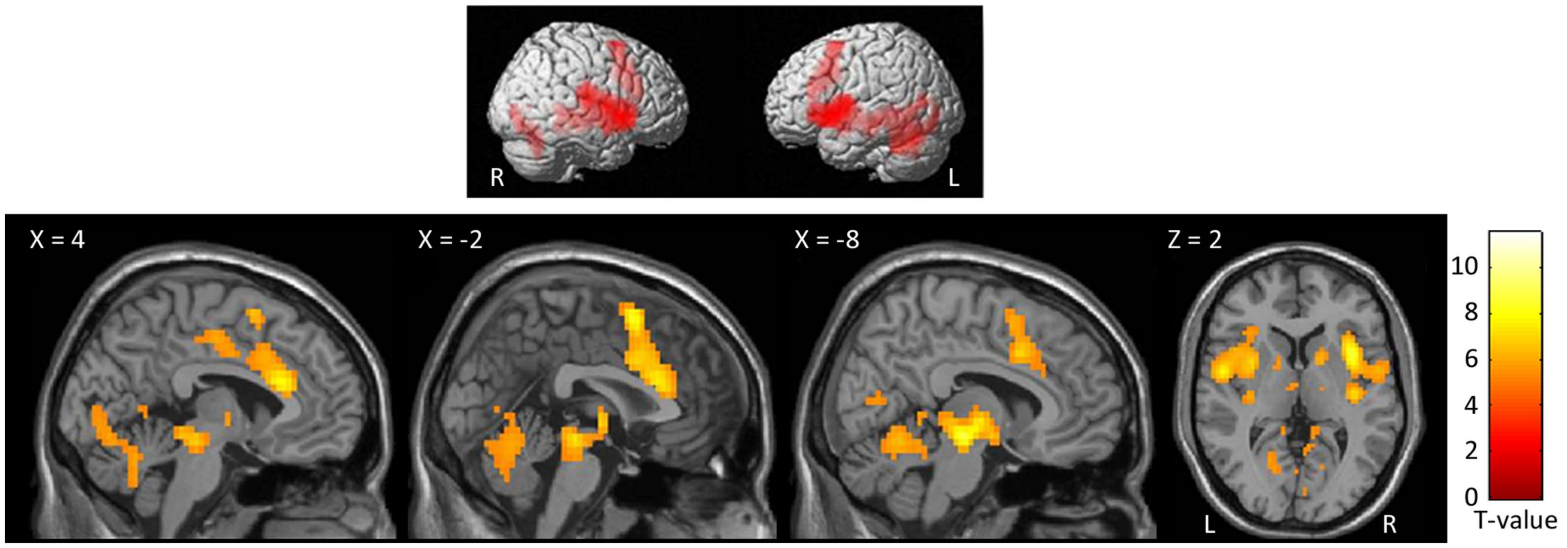
Second level group analyses. Brain areas activation associated with the contrast NocS > NonNocS. Activations are overlaid on the SPM12 canonical template. Xyz coordinates are expressed in MNI. FWE corrected. Color bar represents T-values. NocS, nociceptive stimulation; NonNocS, non-nociceptive stimulation; L, left; R, right.

At the whole-brain level, in the contrast NocS > NonNocS we observed clusters of activation in the right SI and left SII, in the aMCC, in the insula bilaterally, in the inferior frontal gyrus bilaterally, in the left supplementary motor area, and in subcortical structures such as the periaqueductal gray (PAG), thalamus, and basal ganglia (see Figure 3; Table 1). The opposite contrast, NonNocS > NocS, did not reveal any clusters meeting the adopted statistical threshold.

**Table 1:** Regions of increased signal for the contrast NocS > NonNocS.

Brain areas	Side	*k*	*Z*	MNI coordinates		
				*x*	*y*	*z*
Insula, superior temporal gyrus, inferior frontal gyrus, parietal operculum	R	537	7.25	36	−16	14
			6.47	36	11	2
			5.54	57	11	−6
Midbrain (PAG), parahippocampal gyrus, cerebellum, lingual gyrus, thalamus, hippocampus, right basal ganglia	L/R	1,108	6.27	12	−19	−6
			6.25	−12	−22	−10
			5.85	6	−34	−6
Insula, inferior frontal gyrus, superior temporal gyrus	L	432	6.14	−42	14	−2
			5.84	−54	−1	6
			5.70	−33	5	10
aMCC, left supplementary motor area, superior frontal gyrus	L/R	321	5.96	−3	8	58
			5.91	0	23	22
			5.52	−6	11	38
Posterior insula	L	87	5.91	−36	−19	18
Cerebellum	R	27	5.04	33	−58	−30
	R	52	4.86	6	−13	46
Basal ganglia	L	15	4.71	−15	8	2
Supramarginal gyrus	R	9	4.68	63	−22	22
Primary motor cortex, SI	R	12	4.65	36	−25	62
	R	20	4.64	15	−67	10
	R	8	4.55	6	−55	6
SII	L	2	4.45	−54	−16	14

FWE corrected. The ‘MNI coordinates’ column reports coordinates of statistically significant peaks in each cluster, with the highest statistical significances given first. The ‘Brain areas’ column reports all activated areas in the same cluster. L, left; R, right.

#### 3.2.2. Semantic session

##### 3.2.2.1. Physical pain-related words vs. negative non pain-related words

**Figure 4:**
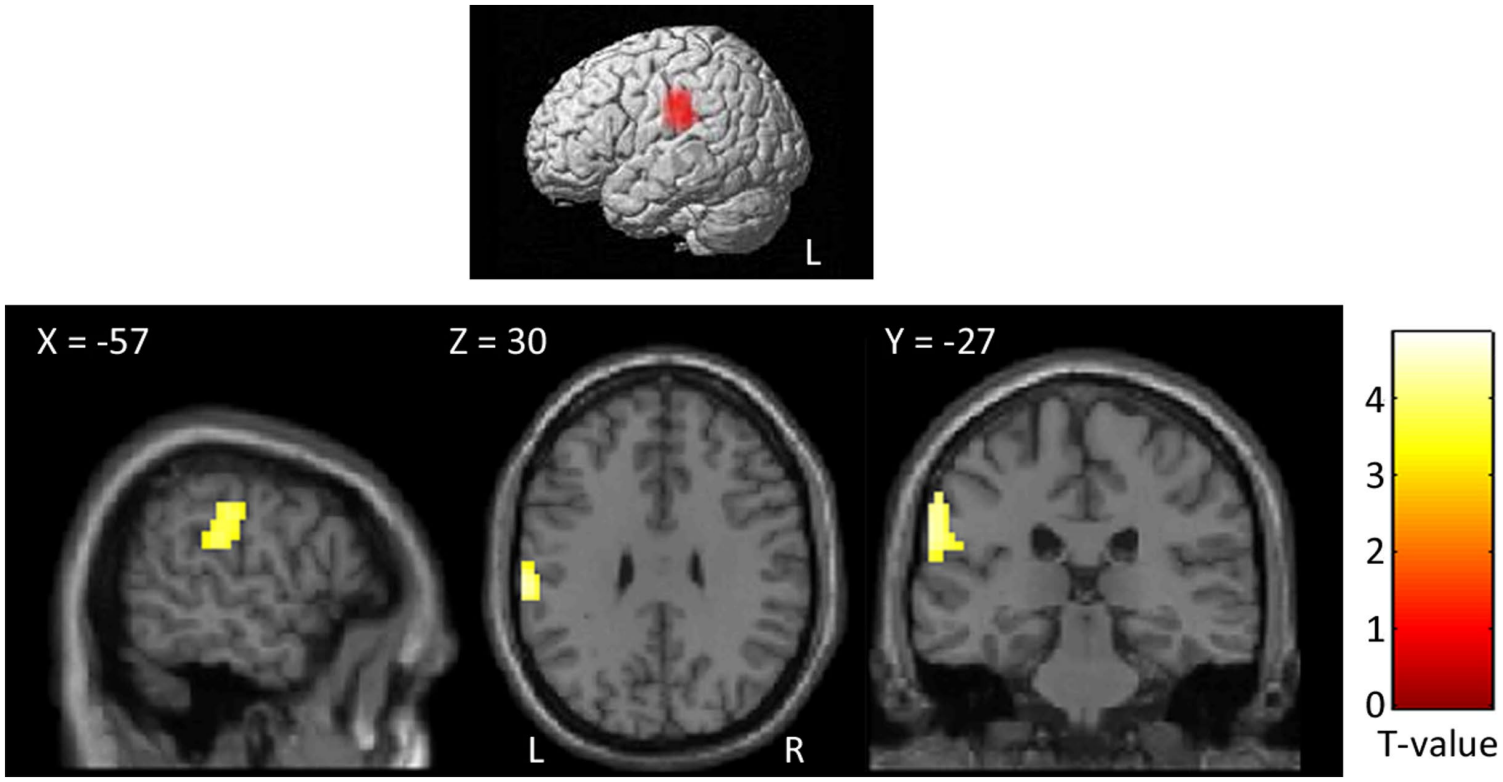
Second level group analyses. Brain areas activation associated with the contrast PhysPW > NegNoPainW. Activations are overlaid on the SPM12 canonical template. Xyz coordinates are expressed in MNI. Double statistical threshold to correct for multiple comparisons: single-voxel statistics *p* < 0.001 and spatial extent > 47, combined α < 0.05. Color bar represents T-values. PhysPW, physical pain-related words; NegNoPW, negative pain-unrelated words; L, left; R, right.

At the whole-brain level, in the contrast PhysPW > NegNoPainW we observed a single cluster of activation encompassing the left supramarginal gyrus, extending to the postcentral gyrus and the superior temporal gyrus (see Figure 4; Table 2). The opposite contrast, NegNoPainW > PhysPW, did not show any clusters meeting the adopted statistical threshold.

**Table 2:** Regions of increased signal for the contrast PhysPW > NegNoPainW.

Brain areas	Side	*k*	*Z*	MNI coordinates		
				*x*	*y*	*z*
Supramarginal gyrus, SI, superior temporal gyrus	L	98	4.18	−63	−22	34

Double statistical threshold to correct for multiple comparisons: single-voxel statistics *p* < 0.001 and spatial extent > 47, combined α < 0.05. The ‘MNI coordinates’ column reports coordinates of statistically significant peaks in each cluster, with the highest statistical significances given first. The ‘Brain areas’ column reports all activated areas in the same cluster. L, left.

##### 3.2.2.2. Social pain-related words vs. negative non pain-related words

**Figure 5:**
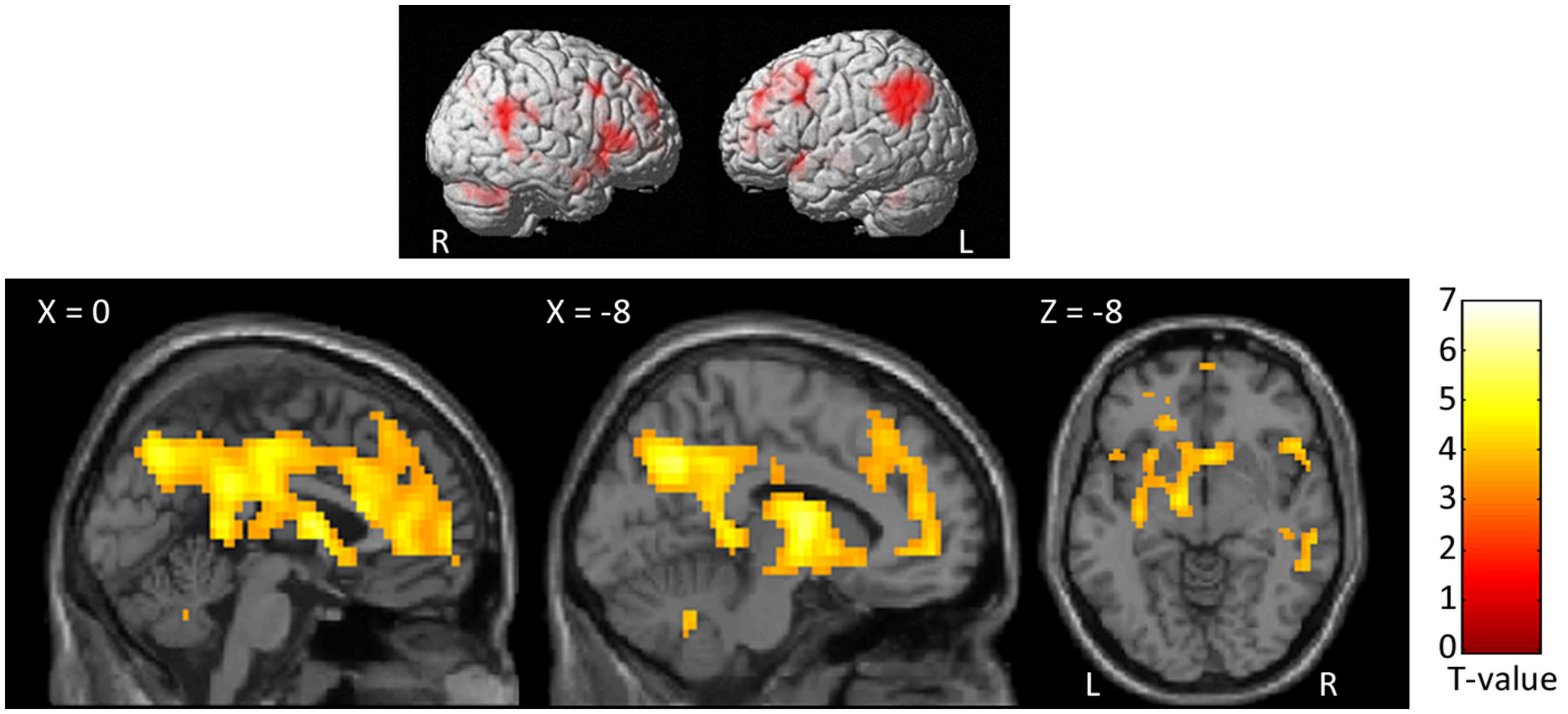
Second level group analyses. Brain areas activation associated with the contrast SocPW > NegNoPW. Activations are overlaid on the SPM12 canonical template. Xyz coordinates are expressed in MNI. Double statistical threshold to correct for multiple comparisons: single-voxel statistics *p* < 0.001 and spatial extent > 63, combined α < 0.05. Color bar represents T-values. SocPW, social pain-related words; NegNoPW, negative pain-unrelated words; L, left; R, right.

At the whole-brain level, in the contrast SocPW > NegNoPW we observed clusters of activation bilaterally in the prefrontal cortex, posterior and ACC, insula, precuneus, thalamus, angular gyrus, supramarginal gyrus, caudate nucleus, middle temporal gyrus, hippocampus, and cerebellum. In the left hemisphere, the activations encompassed the inferior and superior parietal lobules, cuneus and basal ganglia. In addition, we observed activity in the right superior temporal gyrus (see Figure 5; Table 3). The opposite contrast, NegNoPW > SocPW, did not reveal any significant clusters.

**Table 3:** Regions of increased signal for the contrast SocPW > NegNoPW.

Brain areas	Side	*k*	*Z*	MNI coordinates		
				*x*	*y*	*z*
Angular gyrus, inferior parietal gyrus, superior temporal gyrus, middle temporal gyrus, supramarginal gyrus, precuneus	L	566	5.42	−42	−64	46
			4.66	−36	−58	30
			4.51	−48	−52	42
Medial frontal gyrus, superior frontal gyrus, ACC, MCC and posterior cingulate cortex, precuneus, basal ganglia, thalamus, insula, inferior frontal gyrus (pars orbitalis), left hippocampus, left cuneus	L/R	3,808	4.78	12	−7	14
			4.71	−9	−7	10
			4.70	−6	−64	38
Superior temporal gyrus, middle temporal gyrus, inferior parietal lobule, angular gyrus, supramarginal gyrus	R	340	4.53	60	−52	22
			4.16	48	−46	18
			4.03	42	−49	10
Inferior frontal gyrus (pars triangularis and orbitalis), middle temporal gyrus, superior temporal gyrus, insula, hippocampus	R	316	4.25	54	23	10
			3.92	51	38	2
			3.83	48	20	−10
Superior frontal gyrus, middle frontal gyrus, inferior frontal gyrus (pars triangularis and pars opercularis)	L	267	4.24	−39	11	46
			3.91	−27	14	58
			3.89	−30	8	30
Cerebellum	R	315	4.17	27	−52	−34
			4.06	42	−58	−38
			3.88	12	−55	−34
Cerebellum	L	81	3.97	−24	−52	−34
			3.50	−9	−55	−30

Double statistical threshold to correct for multiple comparisons: single-voxel statistics *p* < 0.001 and spatial extent > 63, combined α < 0.05. The ‘MNI coordinates’ column reports coordinates of statistically significant peaks in each cluster, with the highest statistical significances given first. The ‘Brain areas’ column reports all activated areas in the same cluster. L, left; R, right.

#### 3.2.3. Comparison between physical and semantic pain

##### 3.2.3.1. Qualitative and quantitative whole brain comparisons

**Figure 6:**
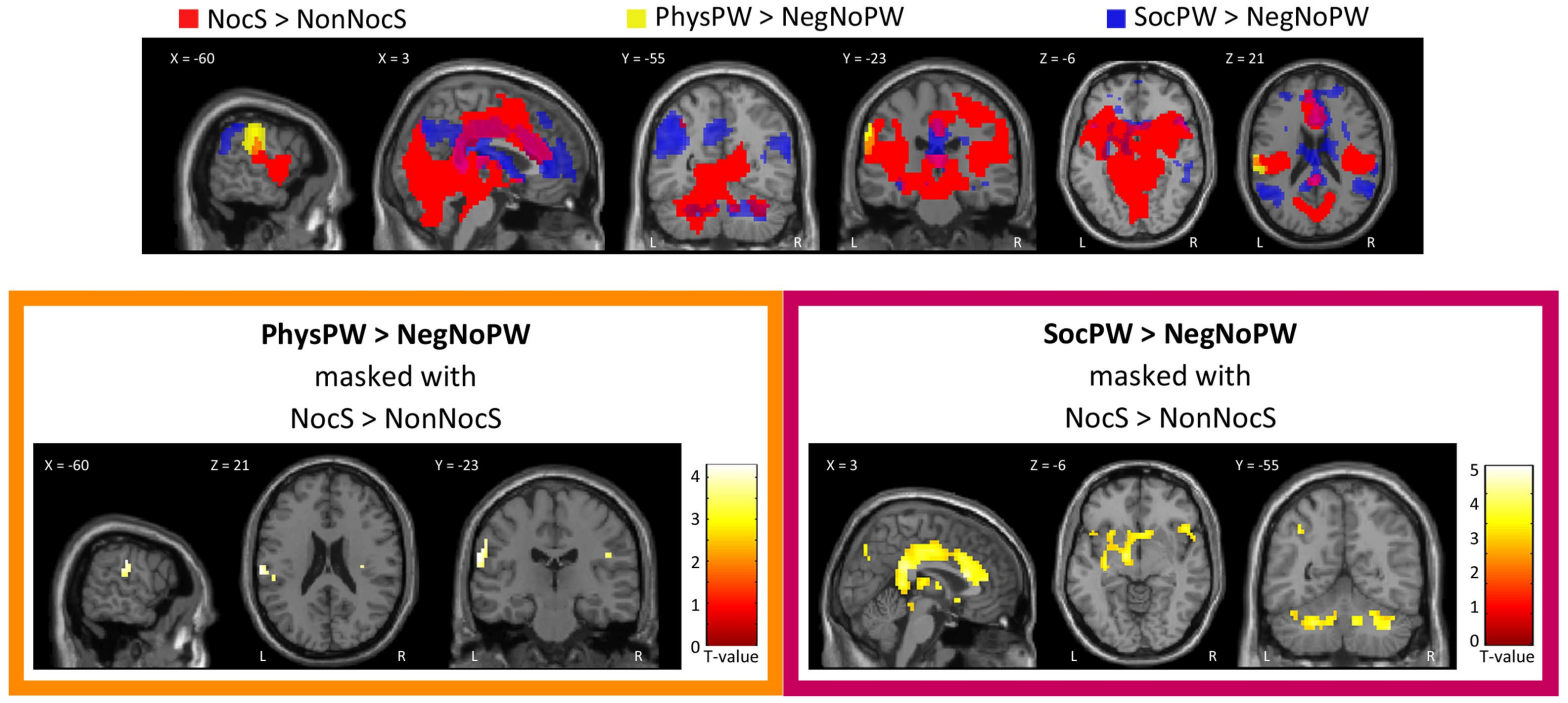
Qualitative (top) and quantitative (bottom) comparison between Nociceptive and Semantic sessions. Top: Suprathreshold clusters for the contrasts NocS > NonNocS (red), PhysPW > NegNoPW (yellow), and SocPW > NegNoPW (blue) are shown; the intersection of NocS > NonNocS and PhysPW > NegNoPW is depicted in orange; the intersection of NocS > NonNocS and SocPW > NegNoPW is depicted in violet. Bottom: Results of the contrasts PhysPW > NegNoPW (left) and SocPW > NegNoPW (right) masked inclusively with the contrast NocS > NonNocS (*p* < 0.001, uncorrected for multiple comparisons). Activations are overlaid on the SPM12 canonical template. Xyz coordinates are expressed in MNI. Color bars represent T-values. NocS, nociceptive stimulation; NonNocS, non-nociceptive stimulation; PhysPW, physical pain-related words; SocPW, social pain-related words; NegNoPW, negative pain-unrelated words; L, left; R, right.

Both qualitative and quantitative analyses performed using the functional map of the NocS > NonNocS contrast as a “spatial localizer” for the semantic pain contrast revealed areas of overlap. Specifically, the PhysPW > NegNoPW map overlaps with NocS > NonNocS in a left-lateralized cluster including the postcentral gyrus and the supramarginal gyrus (Figure 6, top and bottom-left; Table 4A). Instead, several shared regions between the NocS > NonNocS and the SocPW > NegNoPW contrasts were found: anterior, middle and posterior cingulate cortex, right inferior frontal and superior temporal gyri, precuneus, AI, thalamus, and cerebellum, bilaterally (Figure 6, top and bottom-right; Table 4B).

**Table 4:** Regions of increased signal for the contrasts **(A)** PhysPW > NegNoPW (double statistical threshold to correct for multiple comparisons: single-voxel statistics *p* < 0.001 and spatial extent > 47, combined α < 0.05) and **(B)** SocPW > NegNoPW (double statistical threshold to correct for multiple comparisons: single-voxel statistics *p* < 0.001 and spatial extent > 63, combined α < 0.05), each masked inclusively with the NocS > NonNocS contrast (*p* < 0.001, uncorrected for multiple comparisons).

Brain areas	Side	k	*Z*	MNI coordinates		
				*x*	*y*	*z*
**(A) PhysPW > NegNoPW masked with NocS > NonNocS**						
Supramarginal gyrus, postcentral gyrus	L	20	4.19	−63	−25	22
**(B) SocPW > NegNoPW masked with NocS > NonNocS**						
ACC, MCC, and posterior cingulate cortex, medial thalamus, AI, basal ganglia	L/R	1,371	5.11	−9	−7	10
			4.46	3	20	18
			4.44	12	−7	10
Precuneus	L/R	120	5.03	−9	−64	38
			4.29	−3	−73	42
			4.07	6	−73	42
Inferior frontal gyrus (pars triangularis), superior temporal gyrus	R	77	4.17	54	23	2
			4.23	48	20	−10
			4.07	57	8	−14
Cerebellum	R	141	4.17	27	−52	−34
			4.05	39	−55	−38
			4.28	12	−55	−34
AI	R	28	4.39	27	17	−14
Cerebellum	L	79	4.37	−24	−52	−34
			3.50	−9	−55	−30
Cerebellum	L	25	4.07	−45	−61	−30

Only clusters with k >20 are reported. The ‘MNI coordinates’ column reports coordinates of statistically significant peaks in each cluster, with the highest statistical significances given first. The ‘Brain areas’ column reports all activated areas in the same cluster. L, left; R, right.

##### 3.2.3.2. ROI analyses

**Figure 7:**
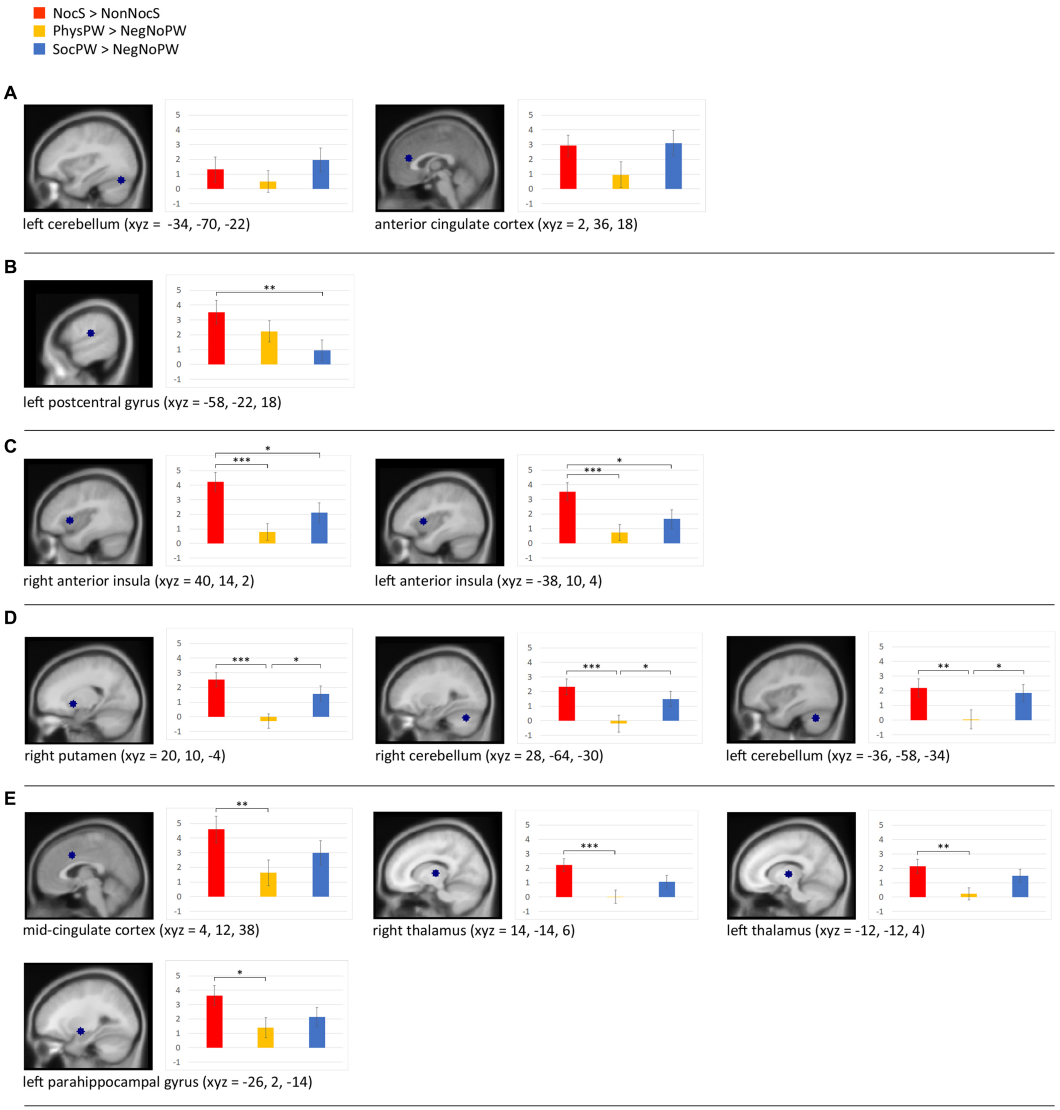
Beta analyses with sagittal section views of the twelve regions of interest (ROIs) and histograms illustrating the beta value means for the stimuli categories NocS (contrast of interest: NocS > NonNocS), PhysPW (contrast of interest: PhysPW > NegNoPW), and SocPW (contrast of interest: SocPW > NegNoPW) and the statistically significant differences: **(A)** brain regions exhibiting no significant differences between word categories; **(B)** brain regions where NocS elicits significantly more activation than SocPW; **(C)** regions where NocS triggers significantly more activation than both PhysPW and SocPW; **(D)** regions where both NocS and SocPW induce significantly higher activation than PhysPW; **(E)** regions where NocS elicits significantly higher activation than PhysPW. Xyz coordinates are expressed in MNI. Error bars represent standard errors. Lines connecting boxplots indicate significant differences with p-values of *<0.05, **<0.01, and ***<0.001. NocS, nociceptive stimulation; NonNocS, non-nociceptive stimulation; PhysPW, physical pain-related words; SocPW, social pain-related words; NegNoPW, negative pain-unrelated words.

The beta analysis we performed on the ROIs obtained by comparing our results with those by Jensen et al. (2016) (see section 2.6.2) revealed (Figure 7): (a) no significant differences between the word categories NocS (contrast NocS > NonNocS), PhysPW (contrast PhysPW > NegNoPW), and SocPW (contrast SocPW > NegNoPW) in one of the two ROIs identified in the left cerebellum (*F* = 1.886, *p* = 0.16), and a significant difference in the ACC (F_Greenhouse-Geisser_ = 3.487, *p* = 0.047), but with none of the subsequent *post hoc* tests reaching the threshold α = 0.05; (b) NocS significantly more activated than SocPW, but no difference with PhysPW, in the left postcentral gyrus (*F* = 5.219, *p* = 0.008); (c) NocS significantly more activated than both PhysPW and SocPW in the right and left AI (*F* = 10.858, *p* < 0.001 and *F* = 9.041, *p* > 0.001, respectively); (d) NocS and SocPW significantly more activated than PhysPW in the right putamen (*F* = 9.407, *p* > 0.001) and in the right and left cerebellum (*F* = 8.603, *p* < 0.001 and *F* = 7.189, *p* = 0.002, respectively); (e) NocS significantly more activated than PhysPW, but no significant differences with SocPW, in the MCC (*F* = 7.048, p = 0.002), right and left thalamus (*F* = 8.597, *p* < 0.001 and F_Greenhouse-Geisser_ = 5.44, *p* = 0.12, respectively), and left parahippocampal gyrus (*F* = 4.013, *p* = 0.023).

#### 3.2.4. Regression and parametric analyses

The regression analyses on the brain activity considering each individual participant’s BIS/BAS and IRI scores did not reveal any significant results.

**Figure 8:**
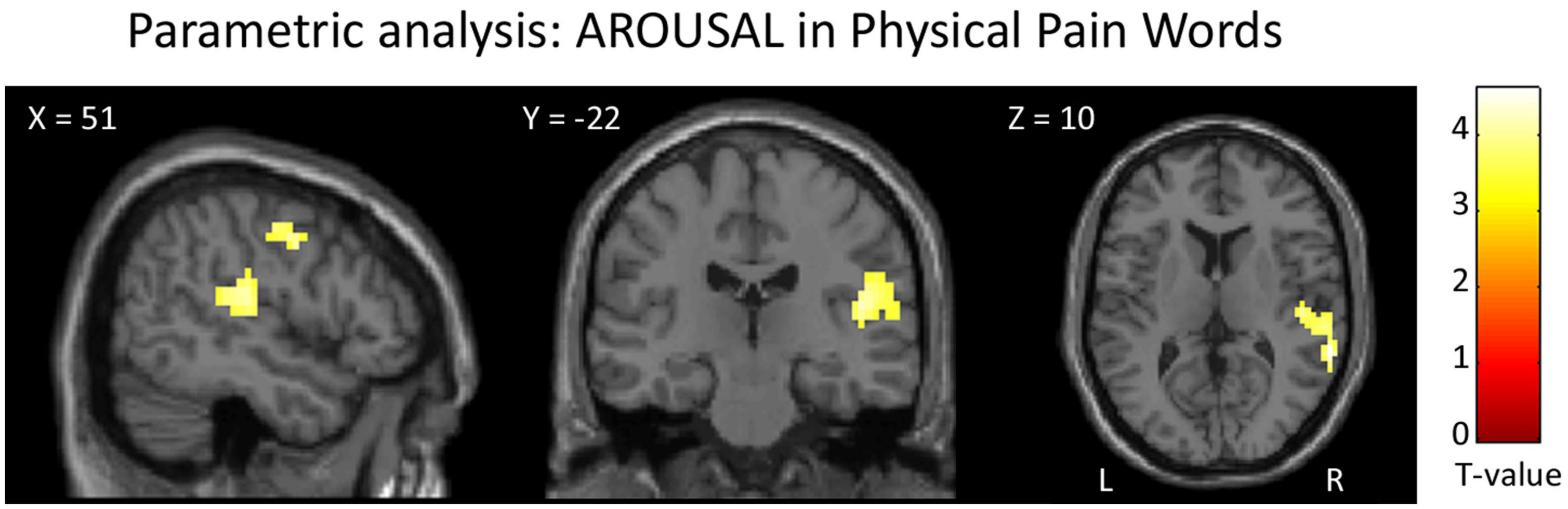
Results of the parametric analysis on the brain activity considering participants’ ratings of arousal as parameters of interest in the analysis of PhysPW. Activations are overlaid on the SPM12 canonical template. Xyz coordinates are expressed in MNI. Double statistical threshold to correct for multiple comparisons: single-voxel statistics *p* < 0.001 and spatial extent > 44, combined α < 0.05. Color bar represents T-values. PhysPW, physical pain-related words; L, left; R, right.

The parametric analysis on brain activity considering participants’ ratings of arousal for each word as parameter of interest in the analysis of PhysPW revealed two significant right-lateralized clusters including the superior and middle temporal gyri, the supramarginal gyrus and the precentral gyrus (Figure 8; Table 5). No significant results were found for other parametric analyses, neither for arousal in other categories of words, nor for participants’ ratings of valence, pain relatedness, intensity, and unpleasantness in any category of words.

**Table 5:** Results of the parametric analysis on the brain activity considering participants’ ratings of arousal as parameters of interest in the analysis of PhysPW.

Brain areas	Side	k	*Z*	MNI coordinates		
				*x*	*y*	*z*
Middle and superior temporal gyrus, supramarginal gyrus, postcentral gyrus, parietal operculum	R	142	4.01	63	−40	10
Pre- and post-central gyrus			4	60	−31	14
			3.70	48	−22	14
	R	47	3.80	54	−10	46
			3.59	48	−4	42

Double statistical threshold to correct for multiple comparisons: single-voxel statistics *p* < 0.001 and spatial extent > 44, combined α < 0.05. The ‘MNI coordinates’ column reports coordinates of statistically significant peaks in each cluster, with the highest statistical significances given first. The ‘Brain areas’ column reports all activated areas in the same cluster. R, right.

## 4. Discussion

The aims of the present study were: (i) to compare the brain areas involved in experiencing nociceptive pain and in processing semantic pain conveyed by physical and social pain-related words in the same individuals; (ii) to clarify whether the processing of semantic pain as conveyed by either physical pain-related words or social pain-related words recruits common or different brain regions; and (iii) to define whether semantic pain activations are linked only to the affective-motivational dimension of pain or also to the sensory-discriminative dimension.

A strength of this study is that, to the best of our knowledge, it is the first one comparing the processing of nociceptive and semantic pain in the same individuals using both words associated with physical and social pain. Overall, the results of this study highlight the presence of an extensive overlap in the areas involved in processing nociceptive and semantic pain. Interestingly, PhysPW and SocPW elicited very different activations: processing SocPW triggered a complex network of neural activity, where the overlap with the nociceptive pain network included activations of the cingulate cortex, anterior insula, medial thalamus, basal ganglia, precuneus, and cerebellum. In contrast, processing PhysPW led to the activation of a much more restricted network where the areas of overlap with the nociceptive pain network included the left supramarginal and postcentral gyrus. In sum, our findings suggest that only the words related to physical pain resonate with areas involved in the sensory-discriminative dimension of pain, whereas words related to social pain resonate with areas involved in the affective-motivational dimension of pain. However, our results also suggest some caution when assigning each area to one dimension or the other of pain processing, as is the case for instance of primary somatosensory cortex (see sections 4.1 and 4.2).

The neural network of pain semantics found in this study is consistent with the brain-based componential semantic representation model of Binder et al. (2016), who identify “Somatic-Pain” (associated with pain or physical discomfort) and “Emotion-Harm” (associated with someone or something that could cause harm) as dimensions of experience that are fundamental for the neural coding of concrete and abstract concepts. A speculative interpretation suggests that the neural activation in pain matrix regions during pain word processing might align with Hebbs’ neural network model (Hebbs, 1949). This model suggests that repeated experiences create a neural memory network. This network strengthens connections and enhances efficacy whenever exposed to similar experiences. In the case of pain, our findings and others’ suggest that the neural activation in pain matrix regions may resonate with prior pain experiences, triggering the associative semantic memory traces of nociceptive pains whenever we face tangible or potential painful stimuli, or stimuli conveying harm or threat (Ritter et al., 2019; Brodhun et al., 2021). Additionally, our results may suggest that the strength of this associative memory network is higher for social pain words than for physical pain words.

### 4.1. Behavioral data and parametric analysis

The words used as experimental stimuli were chosen to be carefully balanced for the main psycholinguistic, distributional, affective, and pain-related variables that are known to affect comprehension processes. To this aim, we used two normative databases (Italian ANEW; Montefinese et al., 2014; WOP; Borelli et al., 2018) which involve large numbers of participants (1,084 and 1,020, respectively). The participants in the present study provided ratings which in part differed from those of the much larger and heterogeneous set of participants tested in the two databases. This was not unexpected, as the sample involved in a normative word corpus development is usually much larger and more heterogeneous compared to the sample involved in a cognitive neuroscience study. In choosing stimuli, whether linguistic or not, researchers have the option of using normative words from databases or collecting them directly from study participants. While normative databases offer standardized measures, ensuring consistency and comparability across studies, they might not capture the nuances of the target population or context. Conversely, collecting normative data directly from study participants is extremely time-consuming yet grants insight into stimuli perception, enhancing their relevance and representativeness of the experimental conditions being studied. Therefore, having integrated norms from word databases with participant’s ratings represents a further strength of our study.

As already mentioned in Methods (section 2.6.2), the stimuli reallocation due to valence differences between our participants’ and normative corpora’ ratings was minimal. Yet, we found that SocPW were significantly more intense and unpleasant than PhysPW. This was not an unexpected result. Although it is undeniable that the two types of pain share common features, their psychological characteristics diverge in many respects. For instance, while feelings of social pain can be re-experienced even years after the painful event (Meyer et al., 2015), the sensory feelings of physical pain cannot be relived after the painful episode. Beyond ours, other studies have found that social pain words are considered to convey more intense and unpleasant painful experiences than physical pain words by both healthy participants and advanced cancer patients (Borelli et al., 2018, 2021a). However, a possible confounding effect of differences in intensity and unpleasantness ratings was ruled out by performing parametric analyses which did not show any significant results for these variables. In fact, the parametric analyses returned only one positive correlation between PhysPW and arousal, in a single cluster including pre- and post-central cortex, superior and middle temporal gyri, and supramarginal gyrus, on the right. The postcentral gyrus, i.e., the somatosensory cortex, is known to respond for actual nociceptive stimulation (Apkarian et al., 2005; Lui et al., 2008; Jensen et al., 2016), although not depending on different pain intensities (Favilla et al., 2014). Yet, the increased activation we found in the postcentral gyrus for the arousal induced by PhysPW suggests that this region may be involved not only in the sensory-discriminative dimension of pain but also in its affective-motivational dimension.

As to the right supramarginal gyrus, together with the adjacent angular gyrus it is part of the inferior parietal lobule, a high level associative brain region which is anatomically and functionally composite. Different authors use different labels to indicate the same areas, or areas which partly overlap with one another, with often ill-defined borders, within this region, e.g., inferior parietal lobule, angular and supramarginal gyri, parietal operculum (e.g., Jensen et al., 2016; Xu et al., 2020). Activation of this complex region is often observed in studies on nociceptive pain, but its specific relevance is not always fully discussed (Jensen et al., 2016). On the other hand, the BOLD signal in the right supramarginal gyrus has been correlated also with different empathic characteristics (Flasbeck et al., 2019; Zhao et al., 2021; Giacomucci et al., 2023) and with emotional regulation competencies (Wadden et al., 2018; Imai et al., 2023), both in healthy and pathological populations. A recent meta-analysis on pain and empathy (Fallon et al., 2020) specifically addressed the possible double involvement of the inferior parietal cortex, identifying a specific role of the more ventral regions of this complex (“parietal operculum”) in sensory functions, whereas the supramarginal gyrus proper, bilaterally, appears involved both in empathy and in pain perception, although with a prevalence for empathy. Our results only partly confirm what was observed by Fallon and colleagues since both the supramarginal gyrus and the parietal operculum were encompassed in our cluster that correlates with arousal in processing PhysPW. These results suggest that both regions may be involved not only in the sensory-discriminative dimension of pain.

Based on our initial stimuli classification through normative databases, we found that PhysPW were significantly more concrete than SocPW, as already found in a previous psycholinguistic study. It has been suggested that abstract concepts are more affectively-laden than concrete concepts (Lenci et al., 2018). The recruitment of the affective-motivational component of pain we have found during the processing of SocPW and not PhysPW may reflect the closer connection of SocPW with abstract concepts. In other words, the different brain activations found for PhysPW and SocPW would mirror the difference between concrete words, more grounded in sensory-motor experiences, and abstract words, primarily grounded in the inner emotional states (Kousta et al., 2011; Meteyard et al., 2012; Vigliocco et al., 2013). However, recent data suggest that the concept of emotional grounding only applies to a limited number of abstract concepts, and that when the measurement of concreteness/abstractness does not rely on concreteness ratings, concrete concepts tend to be rated as more emotional than abstract concepts (Winter, 2023). Therefore, since the concept of the greater emotionality of abstract words is controversial, further testing is needed to investigate whether our results can be explained by this hypothesis.

### 4.2. Overlap between nociceptive pain and each category of pain words

In summary, as expected according to the literature on nociceptive pain (Treede et al., 1999; Apkarian et al., 2005; Lui et al., 2008; Moulton et al., 2010; Xu et al., 2020), in our study NocS enhanced activation in several cortical regions and subcortical structures, involving both the sensory-discriminative and the affective-motivational components of the pain matrix. Reading PhysPW increased the BOLD signal in a cluster encompassing the primary somatosensory cortex and supramarginal gyrus on the left, and this cluster overlaps with the nociceptive pain map. On the other hand, processing SocPW activated both regions that are involved in the processing of pain (Jensen et al., 2016), such as AI, cingulate cortex, medial thalamus, basal ganglia, precuneus, cerebellum, and several other areas that were not identified by the overlap with our spatial localizer (namely: bilateral prefrontal cortex, bilateral supramarginal and angular gyri, bilateral superior and middle temporal gyri, left cuneus and right superior temporal gyrus).

The ROI beta analysis gives us an interesting additional perspective, in that it provides an insight into the different involvement of each brain region in perceiving and discriminating nociceptive pain and the two types of semantic pain presented in this study. Most of these ROIs show the highest signal for nociceptive pain, which is not surprising, considering that the ROIs were selected using the nociceptive pain map as a localizer. However, some interesting differences are apparent.

First of all, the ROI in ACC did not show any significant difference among NocS, PhysPW, and SocPW; this is consistent with a prominent role of this region not just in the sensory dimension of pain, but in the integration of sensory functions and negative affect. Although previous meta-analyses and reviews have often emphasized the role of the ACC in pain perception (Peyron et al., 2000; Apkarian et al., 2005; Lanz et al., 2011; Duerden and Albanese, 2013; Jensen et al., 2016), it is worth noting that what is called ACC or dorsal ACC, often coincide with MCC or aMCC (see specific discussion on this ambiguity in nomenclature, for instance, in Peyron et al., 2000;Vogt, 2016; Rolls, 2019). It is especially interesting to point out that the ROI that falls within aMCC shows a different beta pattern from the ROI in ACC, namely, significantly lower activity for PhysPW, but not for SocPW, as compared to NocS. Our results are consistent with previous studies showing that the aMCC contributes to emotions and/or avoidance behavior (Vogt, 2016; Rolls, 2019) and to the integration of negative affect, pain, and cognitive control (Etkin et al., 2011; Shackman et al., 2011; Spunt et al., 2012), also, with the observation of pain in others (Singer et al., 2004; Vogt, 2016). However, Kragel et al. (2018) found a specialization in aMCC for pain and not for negative affect (including social rejection); nevertheless, we should point out that none of the cited studies used words as stimuli.

The anterior insula bilaterally is the only region which, although being activated by all three categories of stimuli presented in this study, exhibits a significant preference for nociceptive pain over both categories of semantic pain. Therefore, our results point to a notable differentiation between cingulate cortex and anterior insula, the two regions which are often referred to together as involved in the elaboration of the affective dimension of pain, in that the insula appears as more connected to the presence of an actual physical stimulus.

Since the seminal work of Eisenberger et al. (2003; for an overview, see Eisenberger (2015)), many studies reported the activation of part of the affective-motivational component of the pain matrix (i.e., AI and aMCC) when participants experienced or were reminiscent of social pain (Peyron et al., 2000; Eisenberger and Lieberman, 2005; Masten et al., 2012; Cacioppo et al., 2013; Novembre et al., 2015; Rotge et al., 2015). These regions are those most frequently involved in nociceptive pain elaboration (e.g., Lui et al., 2008; for an overview, see Xu et al., 2020), including the codification of pain intensity (Favilla et al., 2014), but also in conditions that include pain modulation, observation of painful stimuli, placebo and nocebo, and empathy (Zaki et al., 2016; Zunhammer et al., 2021; Tu et al., 2022).

In general, AI is involved in the neural processing of negative affect and of self- and other-directed aversive experiences (Lamm and Singer, 2010; Eisenberger, 2012; Čeko et al., 2022). Interestingly, Morese et al. (2019) found that receiving emotional support such as a gentle touch during an experience of social exclusion, compared to informational support like an explanation text, led to a reduction of activity in the right AI. More recently, a comprehensive model of the neural basis of comforting touch has been proposed, including not only comfort from distress and discomfort, but also from pain (Shamay-Tsoory and Eisenberger, 2021).

The left postcentral gyrus is the only ROI among the ones we identified where nociceptive pain induces significantly higher activity than SocPW, but no significant difference with PhysPW. Previous studies using pain-related words (Gu and Han, 2007; Richter et al., 2010; Ritter et al., 2016) showed a rather inconsistent pattern of activations, variably including different portions of the insular and cingulate cortex, secondary somatosensory cortex and other regions, but they never reported enhanced signal in primary somatosensory cortex. This discrepancy may reflect methodological differences among studies: first of all, our experimental sample was much more numerous than in any one of the above-mentioned studies, and it was also homogeneous for the gender of the participants. Furthermore, we only selected unambiguous physical pain words in that none of the PhysPW could be used to denote social pain. In addition, while we only used nouns, other studies used verbs, which necessarily imply actions (Gu and Han, 2007), or adjectives (Richter et al., 2010; Ritter et al., 2016), which, by definition, are modifiers with a semantically large and unspecified content when presented without the nouns they refer to (Palazova et al., 2011). Finally, these previous studies required participants to actively imagine situations connected to the proposed pain-related words, probably eliciting a more vivid emotional representation of the stimuli. It is worth noting that our findings suggest a multifaceted role for the postcentral gyrus, capable of responding not only to the sensory characteristics of nociceptive pain but also to various stimuli semantically related to pain; by the same token, the results of the parametric analysis reveal a correspondence with the arousal induced by pain-related words (see section 4.1); however, it appears that a physical aspect is critical for the response, as words conveying social pain are significantly less effective to this end. The active cluster obtained also included the left supramarginal gyrus. Again, what we pointed out above (see section 4.1) about the somewhat troublesome definition of the inferior parietal lobe, equally applies in the case of the left hemisphere, given the difficulties in precisely defining the anatomical regions and in establishing their functional role. In addition to what has been said above about the right hemisphere, the left inferior parietal lobule is involved in processing pain words (Richter et al., 2010). This region in the left hemisphere is one of the main nodes of the semantic system (Binder et al., 2009; Stoeckel et al., 2009; Huth et al., 2016). We contrasted PhysPW with NegNoPW, therefore the involvement of purely semantic areas should have been ruled out; however, we might hypothesize that the greater engagement of this region was due to a deeper elaboration of words with a greater emotional and/or semantic resonance, compared to more neutral ones.

Several other regions, specifically, aMCC, as already mentioned above, furthermore, right and left thalamus, parahippocampal gyrus, putamen, and cerebellum, bilaterally, had significantly higher signals for NocS than for PhysPW, with the last two showing also significantly higher signals for SocPW than for PhysPW. The involvement of the medial thalamus for the processing of both NocS and SocPW may be due to the involvement of this subcortical region in ascending-to-activate as well as in descending-to-modulate pain pathways (Wang et al., 2016). Since the thalamus is also associated with negative emotions, apprehension, and regulation (Panksepp, 2003; Woo et al., 2014), one possible explanation for the absence of thalamic activation for PhysPW is that they have lower negativity and arousal levels. Traditionally, the cerebellum is associated with motor processing, but several studies reported its involvement in pain (Peyron et al., 2000; Apkarian et al., 2005; Borsook et al., 2008; Moulton et al., 2010) as well as in cognitive process, emotion, and hypnosis (Adamaszek et al., 2017; Santarcangelo and Manzoni, 2021). Although the exact role of this structure in processing pain still is poorly understood, it has been proposed that the cerebellum may integrate affective processing, pain modulation, and sensorimotor processing (Moulton et al., 2010) associated, for instance, to aversive motor responses. Interestingly, our results showed that also SocPW can trigger cerebellar activations as if participants indeed had mentally simulated an aversive response to the words they considered to be the most negative, unpleasant, and arousing.

### 4.3. Limitation and future directions

A possible limitation of this study is that, given the observed gender differences in the perception of physical pain (Dai et al., 2018) and social pain (Benenson et al., 2013; Tomova et al., 2014; Morese et al., 2019), we used an all-female sample (as already done in other studies, e.g., Novembre et al., 2015; Benuzzi et al., 2018) with a narrow age range; however, this choice was made intentionally to identify homogeneous strategies and therefore to increase statistical power. As a future direction, including a more diverse sample in terms of gender and age would provide a more comprehensive understanding of the phenomena under investigation and allow for better generalizability and characterization of the population. Furthermore, since the lack of significant results in the regressions with BIS/BAS and IRI could be due to an inadequate sample size to identify inter-individual differences, future studies should plan a larger sample size and include more scales assessing other personal attitudes toward physical pain and social pain.

Although it could be argued that our acquisition parameters might not be optimal for effectively detecting activation in smaller structures, they were chosen as a compromise between research demands and technical limitations posed by the equipment. Specifically, the decision to employ a voxel size of 3 × 3 × 3 with a 1 mm gap and to include 35 slices was driven by the goal of encompassing the whole brain and the cerebellum; the gap had the additional aim of preventing interference among adjacent slices. The 2-s repetition time (TR) was chosen to accommodate the acquisition of 35 slices. Additionally, the application of a 9x9x12 FWHM Gaussian kernel smoothing was intended to increase the signal-to-noise ratio (e.g., Molloy et al., 2014; Caparelli et al., 2019) and to take into account individual anatomical variations during group analyses.

### 4.4. Conclusion

In summary, we have revealed that, even though the areas involved in experiencing nociceptive pain and processing semantic pain overlap to a great extent, the degree of activity in the various overlapping areas depends on the type of pain conveyed by words. Whereas processing words conveying physical pain appears to activate the postcentral gyrus, a sensory-discriminative area, processing words conveying social pain seems to activate areas associated with the affective-motivational component of pain processing. In most of the regions we analyzed, the signal increase during the processing of words associated with social pain words is not significantly different from that caused by nociceptive stimuli: only in the AIs, activity is significantly higher during nociceptive pain than in both categories of semantic pain.

## Data availability statement

The raw data supporting the conclusions of this article will be made available by the authors, without undue reservation.

## Ethics statement

The studies involving humans were approved by the Ethics Committee of Modena. The studies were conducted in accordance with the local legislation and institutional requirements. The participants provided their written informed consent to participate in this study.

## Author contributions

EBo, CC, and CAP conceptualized the study. EBo, FB, CC, CAP, and FL designed the experiment. EBo, FB, DB, and FL collected the data. EBo, FB, and DB analyzed the data. EBo, FB, DB, CC, and FL contributed to the interpretation of the results and draft the manuscript. EBo, FB, DB, EBa, ML, CC, CAP, and FL provided critical feedback on the manuscript and contributed to the revisions. All authors contributed to the article and approved the submitted version.

## Funding

This research was supported by the FAR2016 grant from the University of Modena and Reggio Emilia (https://www.unimore.it/).

## Conflict of interest

The authors declare that the research was conducted in the absence of any commercial or financial relationships that could be construed as a potential conflict of interest.

## Publisher’s note

All claims expressed in this article are solely those of the authors and do not necessarily represent those of their affiliated organizations, or those of the publisher, the editors and the reviewers. Any product that may be evaluated in this article, or claim that may be made by its manufacturer, is not guaranteed or endorsed by the publisher.

## Footnotes

^1^ http://web.tiscali.it/MarcoSerafini/stimoli_video/

^2^ http://marsbar.sourceforge.net/

^3^ https://afni.nimh.nih.gov/pub/dist/doc/program_help/3dClustSim.html
