# Supplementary material for: Words hurt: common and distinct neural substrates underlying nociceptive and semantic pain
Source: Front Neurosci. 2023 Sep 27;17:1234286. doi: 10.3389/fnins.2023.1234286 (PMC10565001; doi:10.3389/fnins.2023.1234286)
Supplement: Supplementary file 1 [file Data_Sheet_1.docx]

Supplementary Material

|  | PosW | | NegNoPW | | PhysPW | | SocPW | | |
| --- | --- | --- | --- | --- | --- | --- | --- | --- | --- |
|  | Italian | English | Italian | English | Italian | English | Italian | English | |
| Accordo  Ambizione  Asso  Astronauta  Attico  Beatitudine  Brezza  Commedia  Conoscenza  Contante  Coppia  Crepuscolo  Delizia  Diamante  Diploma  Dollaro  Dono Eccitazione  Estasi  Fama  Fascino  Firmamento  Fragranza  Giarrettiera  Godimento  Idolo  Impiego  Incentivo  Infatuazione  Intelletto  Leader  Liberazione  Lusso  Melodia  Milionario  Muffin  Nettare  Nuotatore  Opinione  Orchestra  Orgasmo  Ottimismo  Passione  Patriota  Possibilità  Prateria  Prestigio  Profitto  Villaggio  Trofeo  Trionfo | | Agreement  Ambition  Ace  Spaceman  Penthouse  Bliss  Breeze  Comedy  Knowledge  Cash  Couple  Twilight  Delight  Diamond  Diploma  Dollar  Gift  Excitement  Ecstasy  Fama  Charm  Firmament  Fragrance  Garter  Enjoyment  Idol  Employment  Incentive  Infatuation  Intellect  Leader  Liberation  Luxury  Melody  Millionaire  Muffin  Nectar  Swimmer  Opinion  Orchestra  Orgasm  Optimism  Passion  Patriot  Possibility  Prairie  Prestige  Profit  Village  Trophy  Triumph | Alcolista  Vandalo  Truffa  Collera  Debito  Discarica  Feccia  Ignoranza  Immondizia  Incubo  Minaccia  Miseria  Negligenza  Obesità  Penalità  Tragedia  Terrorista | Alcoholic  Vandal  Fraud  Anger  Debt  Rubbish Arrow  Ignorance  Garbage  Nightmare  Threat  Misery  Negligence  Obesity  Penalty  Tragedy  Terrorist | Artrite  Emicrania  Ferita  Frattura  Frustata  Infarto  Infermità  Infezione  Lacerazione  Lesione  Mutilazione  Nevralgia  Tortura  Piaga  Proiettile  Soffocamento  Ulcera | Arthritis  Migraine  Wound  Fracture  Whip  Heart attack  Infirmity  Infection  Tearing  Wound  Mutilation  Neuralgia  Torture  Plague  Bullet  Suffocation  Ulcer | Abbandono  Aborto  Abuso  Addio  Crepacuore  Depressione  Disperazione  Fallimento  Infelicità  Lacrima  Lutto  Patimento  Perdita  Separazione  Strazio  Stress  Suicidio | | Abandonment  Miscarriage  Abuse  Goodbye  Heartbreak  Depression  Despair  Failure  Unhappiness  Tear  Mourning  Suffering  Lost  Separation  Torment  Stress  Suicide |

**Supplementary Table 1**. List of words used in the Semantic session. Abbreviations: PosW = positive words; NegNoPW = Negative pain-unrelated words; PhysPW = physical pain words; SocPW = social pain-related words.

|  |  | Val (-3/+3) | Aro (1-7) | Pain-rel (1-7) | Int (0-100) | Unpl (0-100) |
| --- | --- | --- | --- | --- | --- | --- |
| PosW | mean (SD) | 1.4 (1.3) | 3.6 (2.2) | 1.2 (0.7) | 5.2 (16.1) | 1.4 (1.2) |
| NegNoPW | mean (SD) | -1.7 (0.9) | 3.8 (2.1) | 3.4 (1.9) | 36.8 (31.8) | 49.5 (11.9) |
| PhysPW | mean (SD) | -2 (0.8) | 4 (2) | 5.3 (1.5) | 59.7 (29.3) | 59.5 (10.5) |
| SocPW | mean (SD) | -2.3 (0.8) | 4.6 (2.2) | 5.7 (1.5) | 72.3 (26.9) | 67 (12.5) |

**Supplementary Table 2**. Descriptive statistics of the words ratings of valence, arousal, pain-relatedness, and intensity given by participants after the Semantic session, and of the words ratings of unpleasantness given by participants during the Semantic session, for each category of words (PosW, NegNoPW, PhysPW, and SocPW). Abbreviations: PosW = positive words; NegNoPW = negative pain-unrelated words; PhysPW = physical pain-related words; SocPW = social pain-related words; Val = valence: Aro = arousal; Pain-rel = pain-relatedness; Int = intensity; Unpl = unpleasantness; SD = standard deviation.

| BIS/BAS | | | | |
| --- | --- | --- | --- | --- |
|  | BIS | REW | DRIVE | FUN |
| Mean | 22.3 | 20.9 | 13.4 | 13.3 |
| SD | 3.7 | 2.5 | 2.2 | 2.6 |
| Range | 16-31 | 15-25 | 9-18 | 9-19 |
|  |  |  |  |  |
| IRI | | | | |
|  | FS | EC | PT | PD |
| Mean | 17.4 | 19.3 | 18.8 | 12.2 |
| SD | 4.4 | 4.1 | 3.9 | 4 |
| Range | 6-26 | 11-27 | 8-28 | 2-21 |

**Supplementary Table 3**. Descriptive statistics related to the BIS/BAS and IRI questionnaires. Abbreviations: BAS = behavioral activation system; BIS = behavioral inhibition system, REW = BAS reward responsiveness, DRIVE = BAS drive, FUN = BAS fun seeking. IRI: FS = fantasy, EC = empathic concern, PT = perspective taking, PD = personal distress.


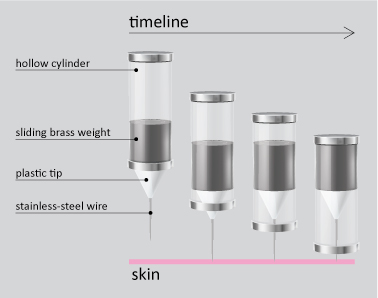


**Supplementary Figure 1.** The figure illustrates the operational mechanism of mechanical stimulation. The aluminum hollow cylinders (here shown transparently for clarity) containing the sliding brass weight, allowed its plastic tip and stainless-steel wire to protrude when held in a vertical position. The experimenter positioned the hollow cylinder with the stainless-steel wire about 1 centimeter from the participant’s hand. During each stimulation, triggered by an LED at predetermined moments, the experimenter gently lowered the hollow cylinder onto the hand. The plastic tip made contact with the skin, causing the sliding brass weight within the hollow cylinder to lift. This resulted in the transfer of its weight onto the stainless-steel wire; therefore, the force with which the tip pressed the skin only depended on the mass of the brass cylinder and on gravity. Once the sliding brass weight was fully retracted into the hollow cylinder, the experimenter lifted the device back to its initial position. Notably, the figure depicts nociceptive stimulation for simplicity; however, tactile, non-nociceptive stimulation was identical except for the use of a foam-rubber tip instead of the stainless-steel wire.
